# Supplementary material for: A fragment-based approach to assess the ligandability of ArgB, ArgC, ArgD and ArgF in the L-arginine biosynthetic pathway of Mycobacterium tuberculosis
Source: Comput Struct Biotechnol J. 2021 Jun 4;19:3491–506. doi: 10.1016/j.csbj.2021.06.006 (PMC8220418; doi:10.1016/j.csbj.2021.06.006)
Supplement: Supplementary data 1 [file mmc1.docx]

**Supplementary information**

**A Fragment-based approach to assess the ligandability of ArgB, ArgC, ArgD and ArgF in the L-arginine biosynthetic pathway of *Mycobacterium tuberculosis***

Pooja Gupta^1^, Sherine E. Thomas^1^, Shaymaa A. Zaidan^2^, Maria A. Pasillas^2^, James Cory-Wright^1^, Víctor Sebastián-Pérez^1,3^, Ailidh Burgess^1^, Emma Cattermole^1^, Clio Meghir^1^, Chris Abell^4^, Anthony G. Coyne^4^, William R. Jacobs Jr. ^5^ Tom L. Blundell^1^, Sangeeta Tiwari^2^* and Vítor Mendes^1#^*.

1 Department of Biochemistry, University of Cambridge, 80 Tennis Court Road, Cambridge, CB2 1GA, UK

2 Department of Biological Sciences & Border Biomedical Research Centre, University of Texas at El Paso, El Paso, TX 79968, USA.

3 Centro de Investigaciones Biológicas Margarita Salas (CSIC), Ramiro de Maeztu 9, 28040 Madrid, Spain.

4 Yusuf Hamied Department of Chemistry, University of Cambridge, Lensfield Road, Cambridge, CB2 1EW, UK

5 Department of Microbiology and Immunology, Albert Einstein College of Medicine, Bronx, NY 10461, USA

# Current affiliation: MRC-Laboratory of Molecular Biology, Molecular Immunity Unit, Francis Crick Ave, Cambridge, CB2 0QH, UK.

* To whom correspondence should be addressed

Vitor Mendes: [vgm23@cam.ac.uk](mailto:vgm23@cam.ac.uk); +44 1223267723

Sangeeta Tiwari: [stiwari@utep.edu](mailto:stiwari@utep.edu); +1 9157476889

**Methods**

**Table S1:** Buffers used for protein purification and storage

| Protein | Buffer A | Buffer B | Buffer C | Storage buffer |
| --- | --- | --- | --- | --- |
| ArgB | 25 mM HEPES pH 7.5  500 mM NaCl  20 mM imidazole | 25 mM HEPES pH 7.5  500 mM NaCl  500 mM imidazole | 25 mM HEPES pH 7.5  200 mM NaCl | |
| ArgC | 20 mM Tris-HCl pH 7.4  500 mM NaCl  20 mM imidazole | 20 mM Tris-HCl pH 7.4  500 mM NaCl  500 mM imidazole | 20 mM Tris-HCl  pH 7.4  500 mM NaCl | 5 mM Tris-HCl  pH 7.4  50 mM NaCl |
| ArgD |  |  | 50 mM Tris-HCl pH 7  100 mM NaCl | |
| ArgF | 50 mM Sodium Phosphate pH 7.4  500 mM NaCl  20 mM imidazole | 50 mM Sodium Phosphate pH 7.4  500 mM NaCl  500 mM imidazole | 50 mM Sodium Phosphate pH 7.4  100 mM NaCl | |

**Results**


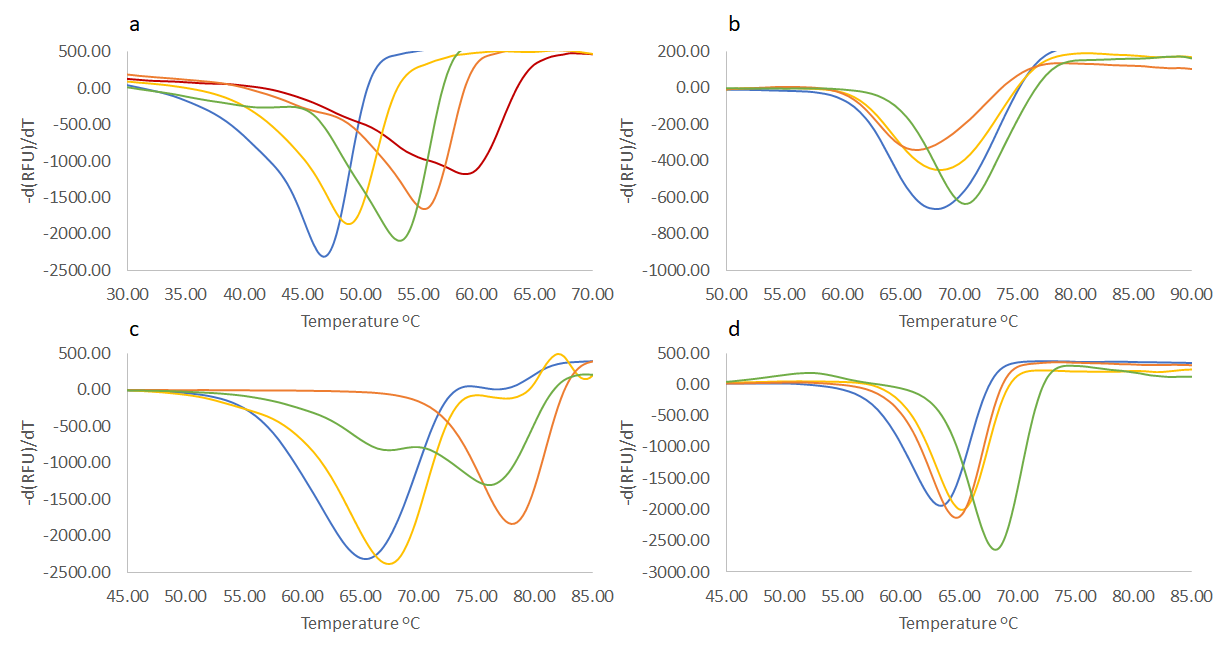


**Figure S1**: (a) DSF profiles of ArgB apo form (blue), in the presence of 1 mM ATP (yellow), 1 mM NAG (orange), 1 mM arginine (red) and a representative fragment hit (green). (b) DSF profiles of ArgC apo form (blue), in the presence of 1 mM NADP^+^ (yellow), 1 mM NADPH (orange) and a representative fragment hit (green). (c) DSF profiles of ArgD showing PLP-unsaturated ArgD (blue), in the presence of 1 mM PLP (orange), and in the presence of two different fragments (yellow and green). PLP-unsaturated ArgD presents two melting peaks, the largest one representing the protein populations without PLP-bound in the dimer or with only protomer containing PLP. The smaller peak has similar melting temperature to the PLP saturated enzyme and likely represents a population where both protomers contain PLP. (d) DSF profiles of ArgF apo form (blue), in the presence of 1 mM L-ornithine (yellow), 1 mM L-citruline (orange) and a representative fragment hit (green).


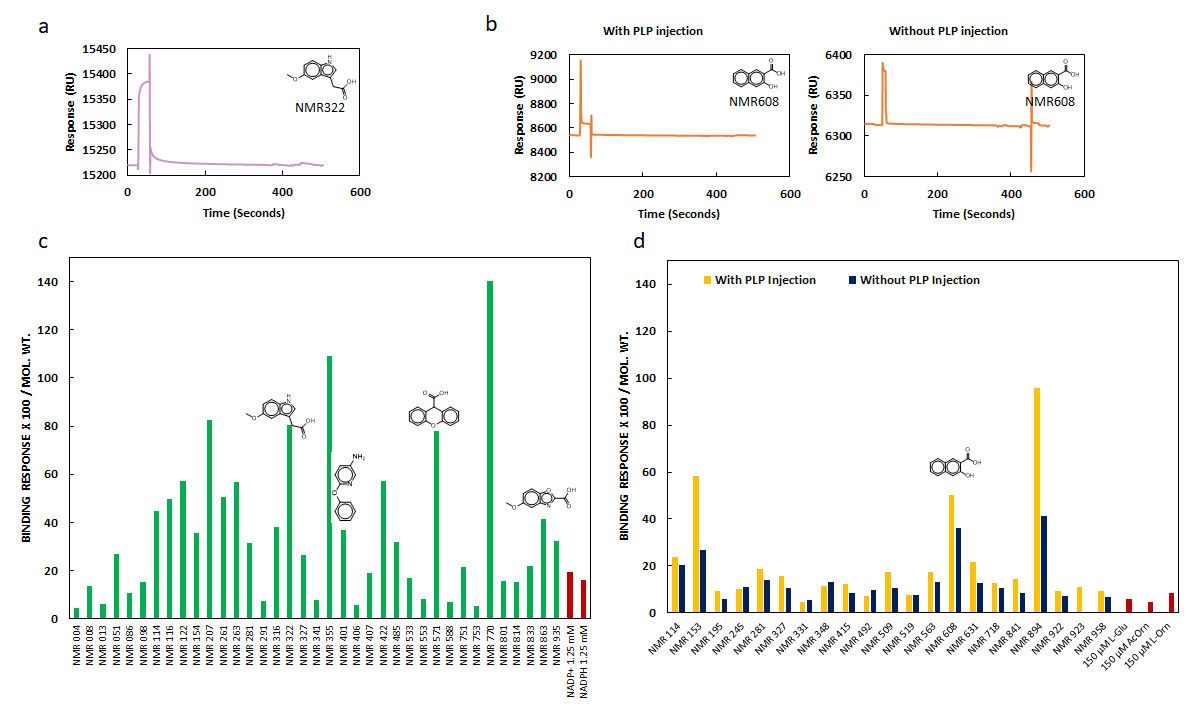


**Figure S2:** (a) and (b) Representative SPR sensorgrams for ArgC and ArgD. (c) Molecular weight-adjusted SPR binding response of DSF fragment hits (tested at 1 mM concentration) against ArgC, along with the positive controls NADP^+^ and NADPH (d) Molecular weight-adjusted SPR binding response of DSF fragment hits (tested at 1 mM concentration) from both screening experiments against ArgD, along with the positive controls glutamate, N-acetylornithine and L-ornithine.


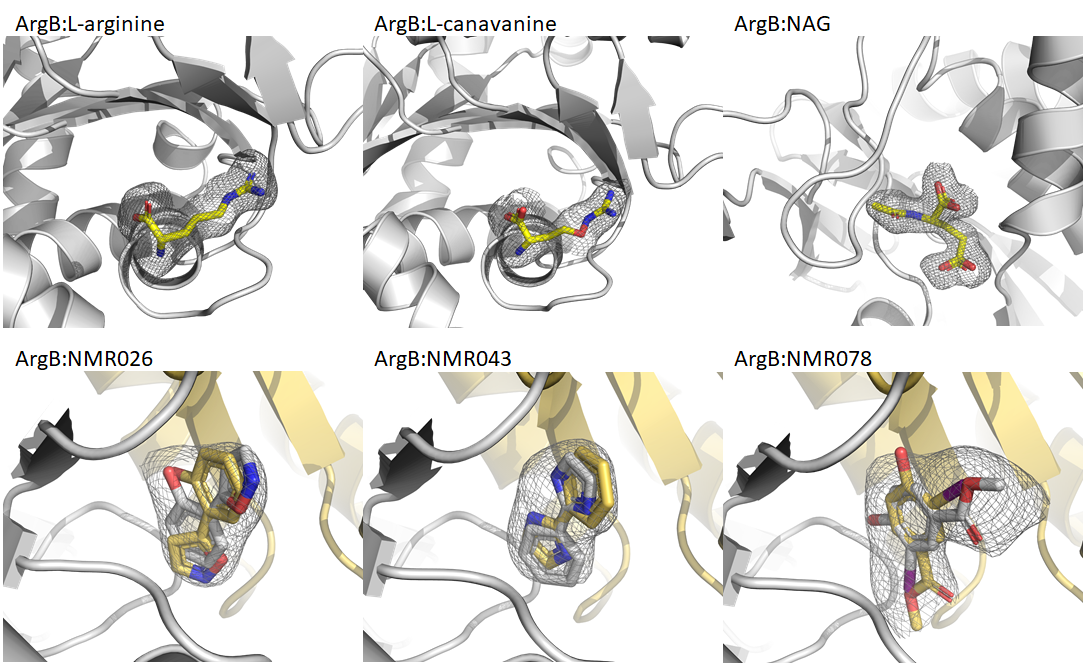


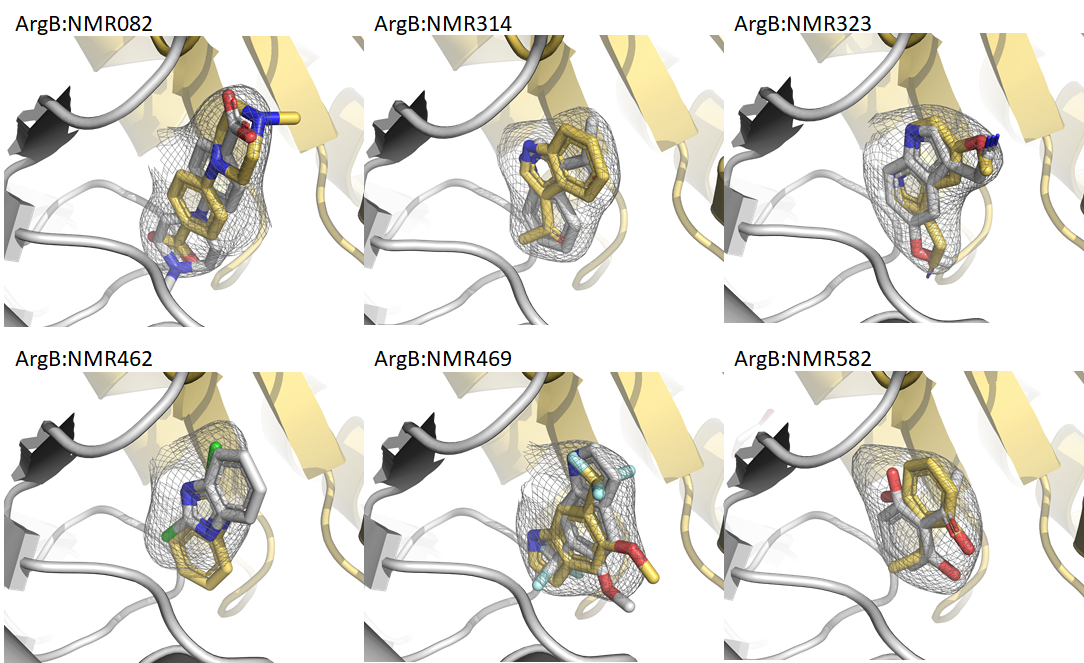


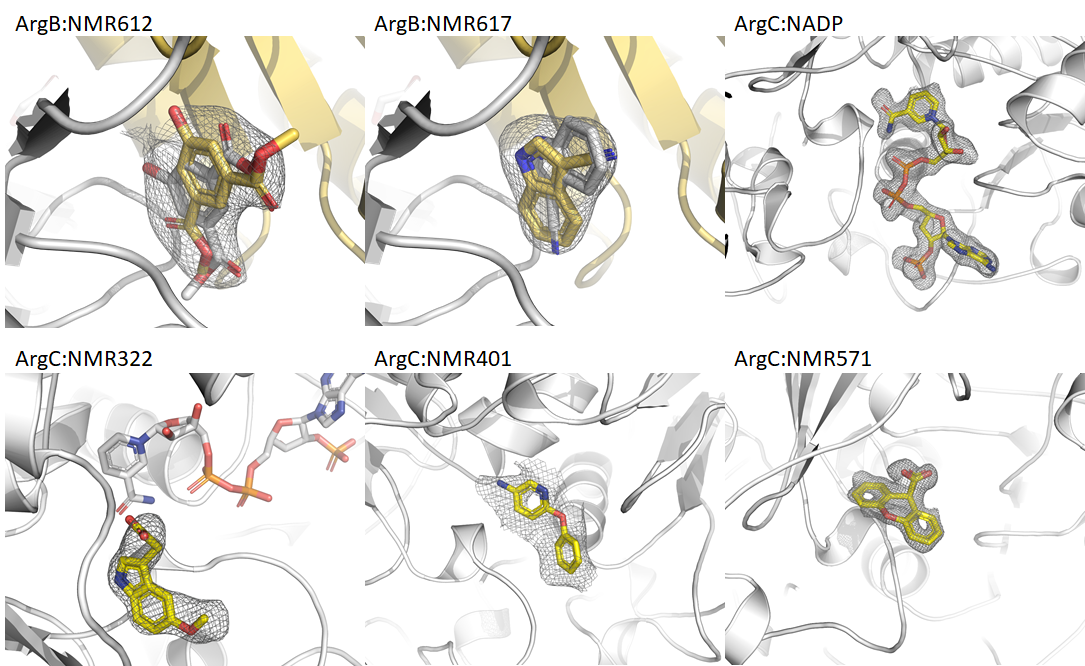


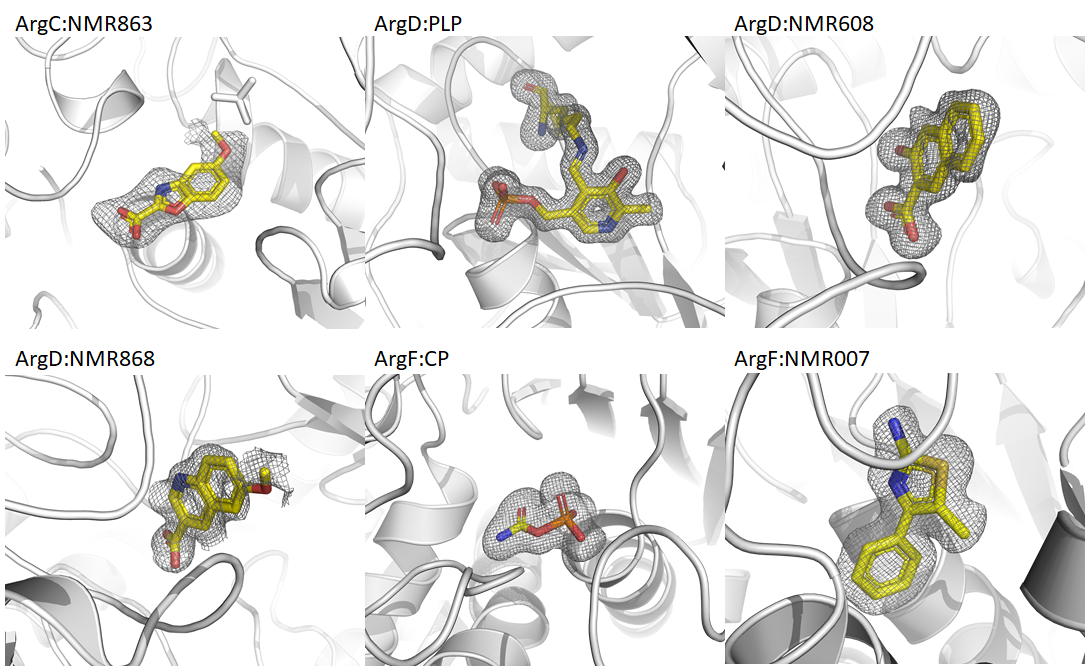


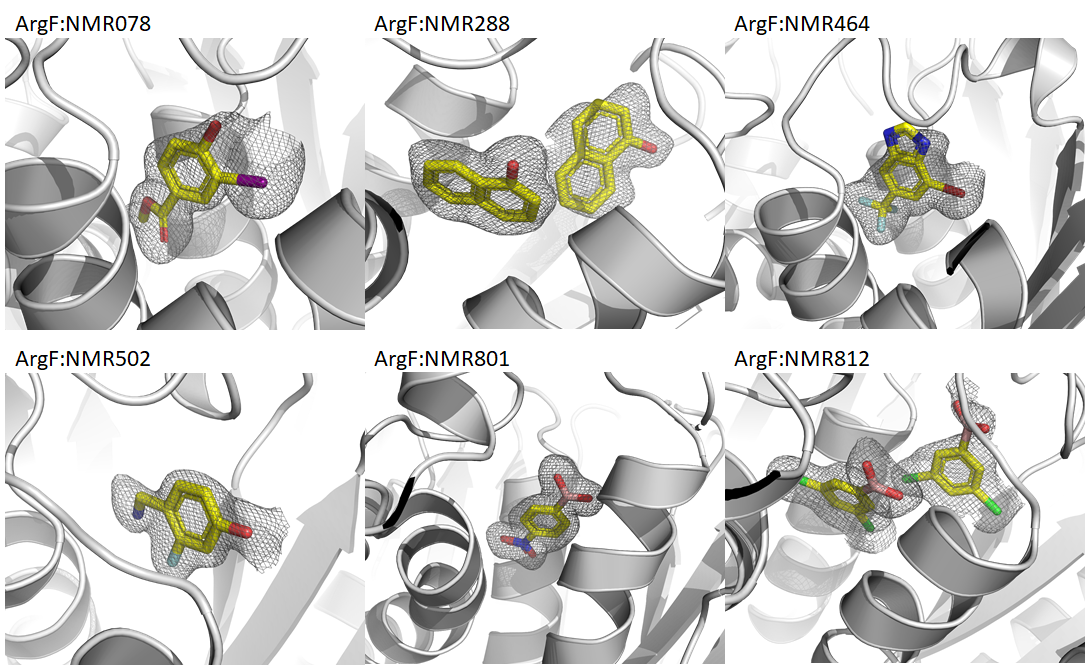


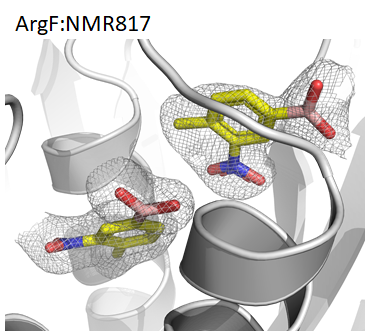


**Figure S3:** [Fo - Fc] “Omit maps” of all ligands identified in this work (except ArgB:NMR711 and ArgB:NMR446 (which are shown in figure 2e) contoured at 1.5σ.


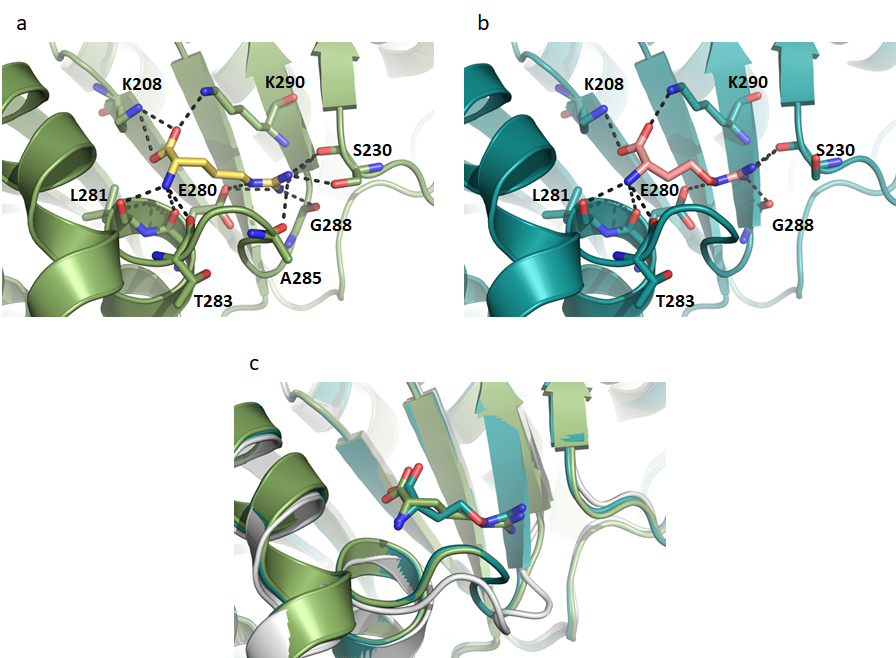


**Figure S4:** X-ray crystal structures of ArgB in complex with L-arginine (a) and L-canavanine (b). Hydrogen bonds between ligands and residues are shown as black dashed lines. (c) Superposition of ArgB Apo structure (white) with ArgB in complex with L-arginine (green) and L-canavanine (blue) illustrating the conformational changes induced by ligand binding. L-arginine and L-canavanine binding to ArgB induce similar conformational changes.


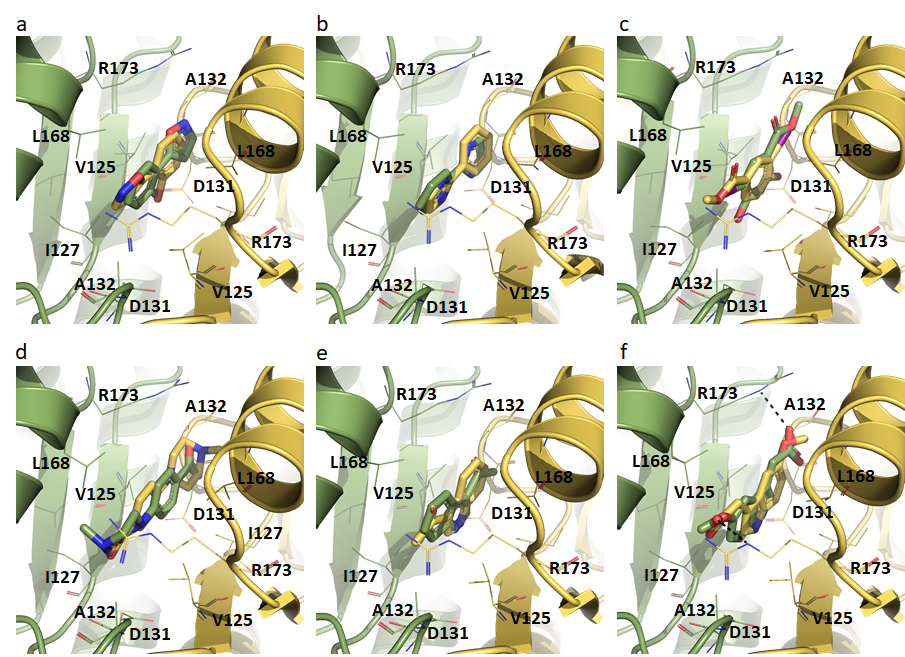


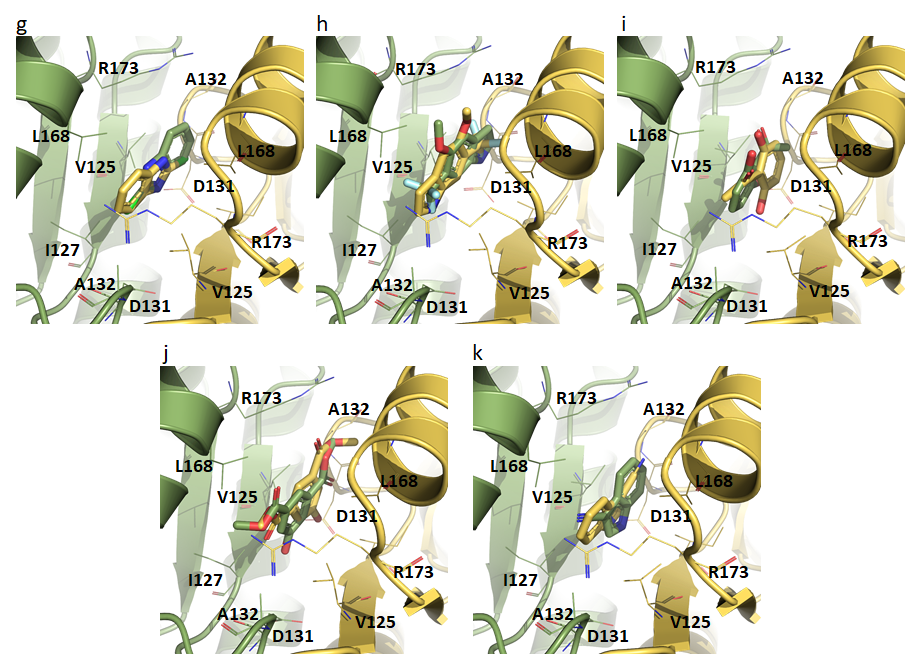


**Figure S5:** X-ray crystal structures of ArgB in complex with compounds NMR026 (a), NMR043 (b), NMR078 (c), NMR082 (d), NMR314 (e), NMR323 (f), NMR462 (g), NMR469 (h), NMR582 (i), NMR612 (j) and NMR617 (k). As the site is symmetrical and sits at a 2-fold crystallographic symmetry axis each fragment exhibits two binding conformations. Each of the binding conformations is in the same colour scheme as the corresponding protomer. Only NMR323 forms an hydrogen bond (depicted as a back dashed line) with the protein (R173 side chain). All other fragments only form hydrophobic and π interactions.


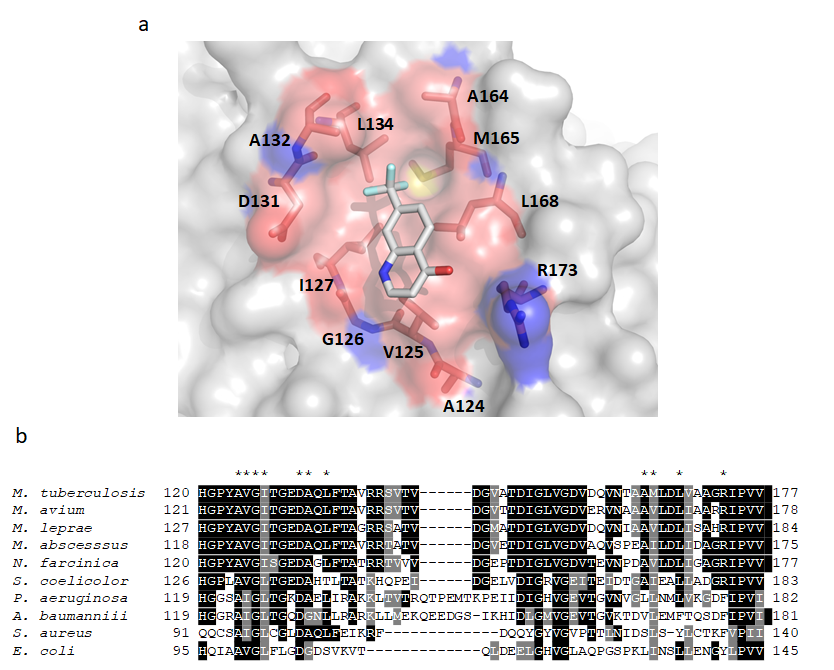


**Figure S6**: (a) Crystal structure of ArgB in complex with NMR446 with interface site residues highlighted by asterisks above the alignment. Only one conformation of the ligand and one protomer are shown for clarity. (b) Alignment of ArgB sequences from *Mycobacterium tuberculosis*, *Mycobacterium avium*, *Mycobacterium leprae*, *Mycobacterium abscessus*, *Nocardia farcinica*, *Streptomyces coelicolor*, *Pseudomonas aeruginosa*, *Acinetobacter baumannii*, *Staphylococcus aureus* and *Escherichia coli* was performed using clustal omega (1). The alignment shows high conservation of residues at the interface site in mycobacteria and closely related species. Non-actinobacterial species (*P. aeruginosa*, *A. baumannii*, *S. aureus* and *E. coli*) show lower conservation. Residues forming this site are highlighted in red. ArgB of *E. coli* and *S. aureus* are dimeric as they lack the N-terminal helix that allows the formation of the hexamer.


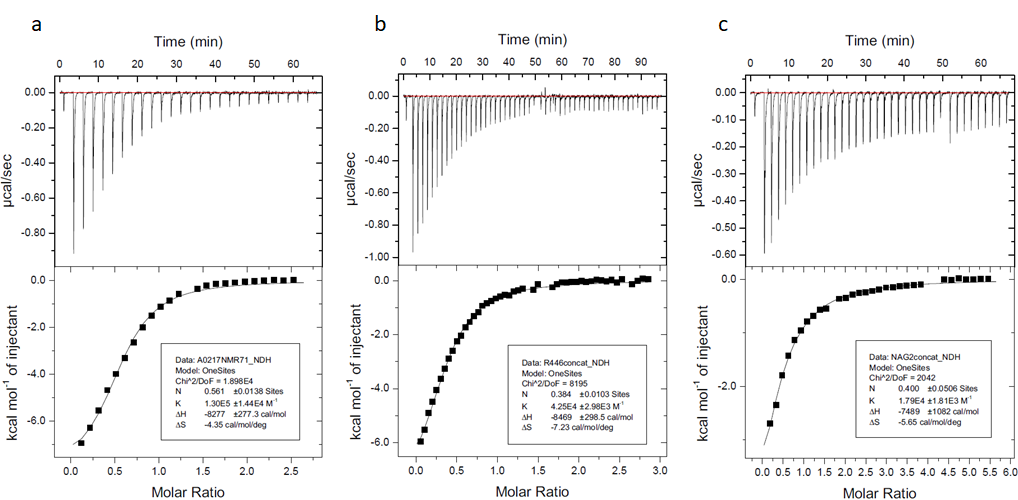

**Figure S7:** ArgB ITC titrations for NMR711 (a), and NMR446 (b), NAG (c), L-arginine (d), and L-canavanine (e).


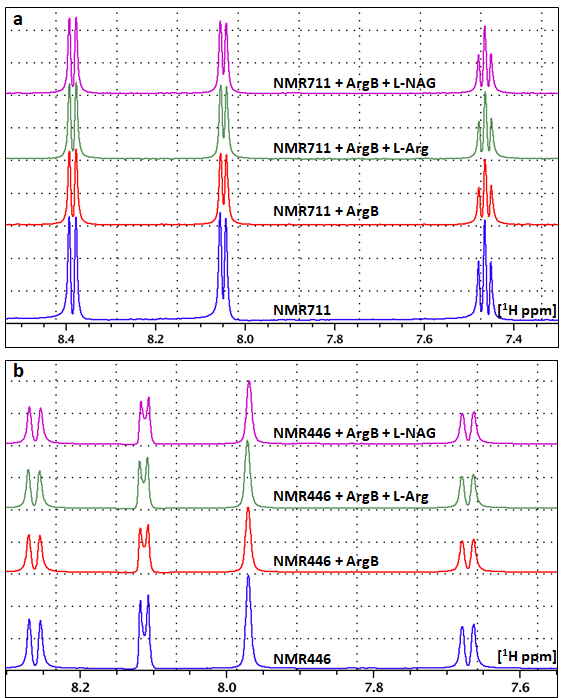


**Figure S8:** Ligand-observed NMR (CPMG) technique to illustrate binding of compounds (a) NMR711 and (b) NMR446 to ArgB. ^1^H NMR spectra of the fragment in the absence (blue) and presence of ArgB (red) are shown, where a drop in overall signal in the presence of ArgB indicates ligand binding to the protein. ^1^H NMR spectra of fragment in the presence L-arginine (green) or L-NAG (magenta) are also overlaid, where no significant change in signal indicates that the fragments bind to an allosteric site.


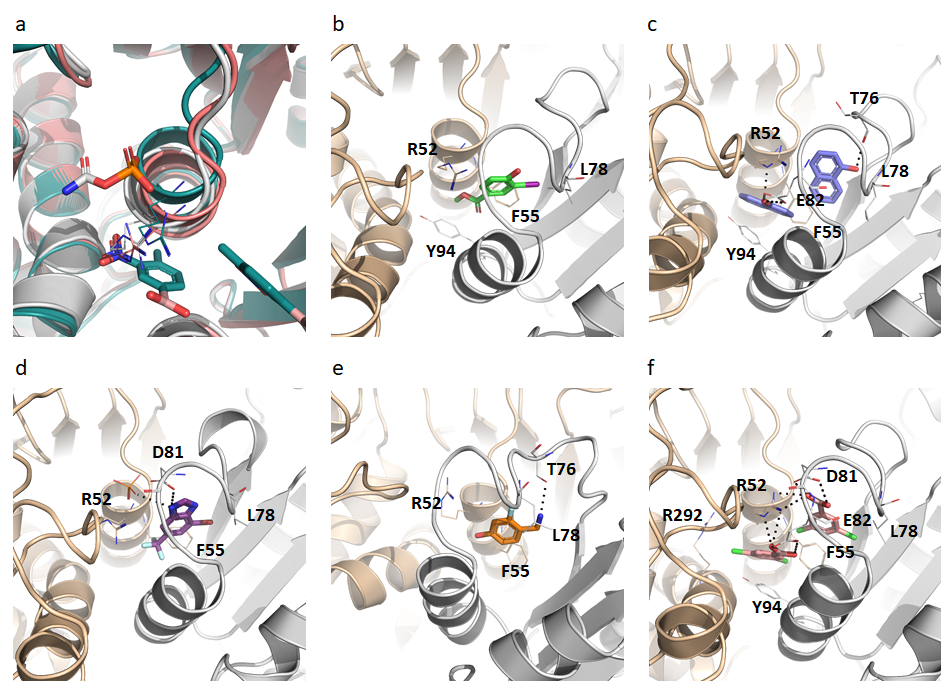


**Figure S9**: (a) Superposition of ArgF apo structure in pink with the carbamoyl phosphate bound structure in white and NMR817 structure in teal, showing movement of helix 2 upon binding of ligands. The distance the α carbon of arg52 is 1.3 Å between the apo and carbamoyl phosphate structures and 1.9 Å between the NMR817 and carbamoyl phosphate structures. X-ray crystal structures of ArgF in complex with compounds NMR078 (b), NMR288 (c), NMR464 (d), NMR502 (e), NMR812 (f). Black dashed lines denote hydrogen bonds. Each protomer of the interfacial site is coloured differently.


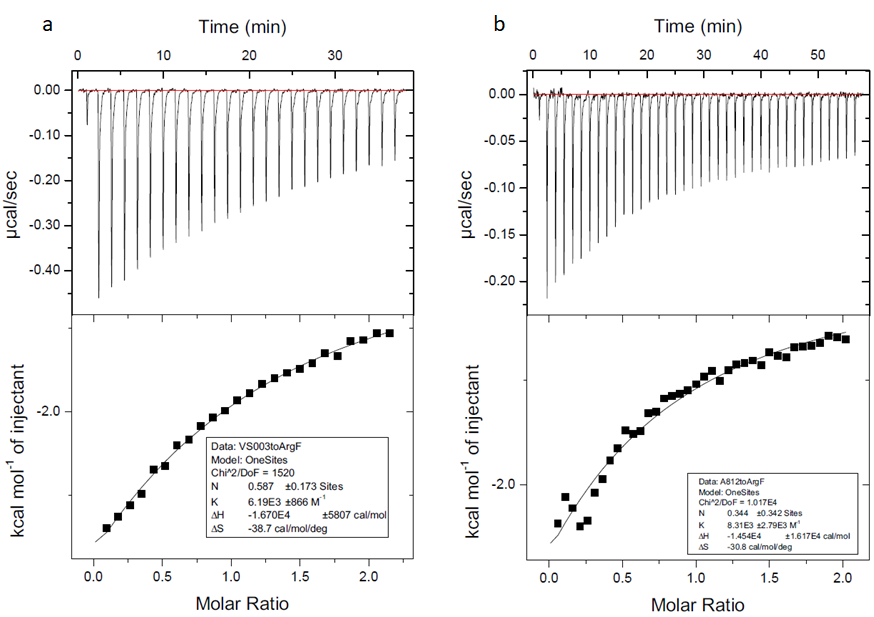


**Figure S10**: ArgF ITC titrations for NMR007 (a) and NMR812 (b).

**Table S2:** ArgB % inhibition at 2.5 mM.

| Compound | Fragment structure | % inhibition |
| --- | --- | --- |
| NMR026 |  | 8 ± 1 |
| NMR043 |  | 5 ± 2 |
| NMR078 |  | 6 ± 1 |
| NMR082 |  | 8 ± 1 |
| NMR314 |  | 6 ± 3 |
| NMR323 |  | 18 ± 2 |
| NMR462 |  | 6 ± 1 |
| NMR469 |  | 0 |
| NMR582 |  | 7 ± 3 |
| NMR612 |  | 11 ± 3 |
| NMR617 |  | 0 |

**Table S3:** X-ray crystallography data collection and final refinement statistics

| Protein | **ArgB** | **ArgB** | **ArgB** | **ArgB** | **ArgB** | **ArgB** | **ArgB** | **ArgB** | **ArgB** |
| --- | --- | --- | --- | --- | --- | --- | --- | --- | --- |
| Ligand# | **APO** | **NAG** | **L-arginine** | **L-canavanine** | **NMR711** | **NMR446** | **NMR026** | **NMR043** | **NMR078** |
| PDB ID | 7NLF | 7NLN | 7NLO | 7NLP | 7NNB | 7NLX | 7NLQ | 7NLR | 7NLS |
| **Data collection*** |  |  |  |  |  |  |  |  |  |
| Space group | *R32* | *P6_3_* | *R32* | *R32* | *R32* | *R32* | *R32* | *R32* | *R32* |
| Cell parameters:  a [Å]  b [Å]  c [Å]  α/β/γ [˚] | 173.95  173.95  70.29  90/90/120 | 100.24  100.24  124.53  90/90/120 | 175.30  175.30  70.54  90/90/120 | 174.06  174.06  71.15  90/90/120 | 173.88  173.88  71.91  90/90/120 | 174.48  174.48  72.54  90/90/120 | 174.93  174.93  72.90  90/90/120 | 173.82  173.82  72.43  90/90/120 | 174.24  174.24  72.03  90/90/120 |
| Resolution range [Å] | 86.98 – 2.08  (2.19 – 2.08) | 86.81 – 1.92  (2.02 – 1.92) | 63.97 – 1.82  (1.92 – 1.82) | 87.03– 2.21  (2.33 – 2.21) | 52.00 – 2.19  (2.30 – 2.19) | 87.24 – 2.23  (2.35 – 2.23) | 65.70 – 2.50  (2.63 – 2.50) | 86.91 – 2.25  (2.39 – 2.25) | 87.12 – 2.65  (2.79 – 2.65) |
| No. of observations  total  unique | 254931  (38396)  25346  (3662) | 566363  (40764)  54154  (7879) | 372764  (54564)  37098  (5393) | 154874  (20116)  20426  (2811) | 195399  (28703)  21590  (3120) | 156940  (20049)  20098  (2605) | 128210  (18834)  14890  (2140) | 199430  (31714)  19795  (3139) | 124133  (18498)  12283  (1774) |
| R_merge_ | 0.045(0.876) | 0.071 (0.595) | 0.041 (0.975) | 0.057 (0.494) | 0.037 (0.787) | 0.041 (0.824) | 0.078 (0.863) | 0.047 (0.816) | 0.072 (1.213) |
| I/σ(I) | 22.2 (2.3) | 19.6 (2.5) | 23.9 (2.6) | 14.6 (2.2) | 27.7 (2.5) | 22.3 (2.2) | 14.4 (2.2) | 23.1 (2.3) | 20.3 (1.9) |
| CC(1/2) | 1.000 (0.885) | 0.999 (0.813) | 0.999 (0.895) | 0.999 (0.951) | 1.000 (0.905) | 0.999 (0.803) | 0.998 (0.747) | 0.999 (0.843) | 0.999 (0.759) |
| Completeness [%] | 100 (99.9) | 99.9 (99.6) | 100 (99.9) | 98.9 (94.0) | 100 (100) | 97.7 (87.3) | 99.8 (99.3) | 99.8 (98.1) | 100 (100) |
| Multiplicity | 10.1 (10.5) | 10.5 (5.2) | 10.0 (10.1) | 7.6 (7.2) | 9.1 (9.2) | 7.8 (7.7) | 8.6 (8.8) | 10.1 (10.1) | 10.1 (10.4) |
| **Refinement** |  |  |  |  |  |  |  |  |  |
| Refinement program | PHENIX | PHENIX | PHENIX | PHENIX | PHENIX | PHENIX | PHENIX | PHENIX | PHENIX |
| Resolution [Å] | 86.98 – 2.08 | 86.81 – 1.92 | 63.97 – 1.82 | 87.03– 2.21 | 52.00 – 2.19 | 87.24 – 2.23 | 65.70 – 2.50 | 86.91 – 2.25 | 87.12 – 2.65 |
| No. reflections | 25346 | 54154 | 37098 | 20426 | 21590 | 20098 | 14890 | 19795 | 12283 |
| R_work_/R_free_ [%] | 18.2/21.6 | 16.5/19.6 | 18.8/20.3 | 20.5/24.1 | 19.9/25.9 | 19.5/23.7 | 18.7/22.5 | 18.4/21.4 | 19.3/25.6 |
| RMS deviations |  |  |  |  |  |  |  |  |  |
| Bonds [Å] | 0.008 | 0.008 | 0.007 | 0.008 | 0.009 | 0.009 | 0.009 | 0.008 | 0.009 |
| Angles [˚] | 0.881 | 1.016 | 0.859 | 1.014 | 0.978 | 0.963 | 0.973 | 0.908 | 1.093 |
| Ramachandran |  |  |  |  |  |  |  |  |  |
| Favoured [%] | 97 | 97 | 97 | 96 | 95 | 97 | 96 | 97 | 95 |
| Outliers [%] | 0.3 | 0.3 | 0.3 | 0.3 | 0.3 | 0.7 | 1.0 | 1.0 | 1.0 |

* Parameters shown in brackets are for the highest resolution shell

**Table S3:** X-ray crystallography data collection and final refinement statistics (cont)

| Protein | **ArgB** | **ArgB** | **ArgB** | **ArgB** | **ArgB** | **ArgB** | **ArgB** | **ArgB** | **ArgC** |
| --- | --- | --- | --- | --- | --- | --- | --- | --- | --- |
| Ligand# | **NMR082** | **NMR314** | **NMR323** | **NMR462** | **NMR469** | **NMR582** | **NMR612** | **NMR617** | **Apo** |
| PDB ID | 7NLT | 7NLU | 7NLW | 7NLY | 7NLZ | 7NM0 | 7NN7 | 7NN8 | 7NNI |
| **Data collection*** |  |  |  |  |  |  |  |  |  |
| Space group | *R32* | *R32* | *R32* | *R32* | *R32* | *R32* | *R32* | *R32* | *C2* |
| Cell parameters:  a [Å]  b [Å]  c [Å]  α/β/γ [˚] | 173.63  173.63  71.99  90/90/120 | 174.49  174.49  71.73  90/90/120 | 174.35  174.35  72.79  90/90/120 | 174.94  174.94  72.96  90/90/120 | 174.06  174.06  72.81  90/90/120 | 174.35  174.35  72.1  90/90/120 | 174.16  174.16  72.06  90/90/120 | 174.65  174.65  72.12  90/90/120 | 140.69  77.99  88.29  90/127.5/90 |
| Resolution range [Å] | 50.12 – 2.25  (2.35 – 2.23) | 64.80 – 2.24  (2.36 – 2.24) | 87.18 – 2.32  (2.45 – 2.32) | 87.47 – 2.25  (2.37 – 2.25) | 87.03 – 2.16  (2.28 – 2.16) | 87.17 – 2.28  (2.40 – 2.28) | 65.02 – 2.17  (2.29 – 2.17) | 87.32 – 2.27  (2.39 – 2.27) | 68.45 – 1.54  (1.63 – 1.54) |
| No. of observations  total  unique | 209659  (30760)  20440  (2950) | 180087  (26623)  20234  (2929) | 157958  (24489)  18318  (2657) | 155612  (23619)  20451  (2982) | 200575  (29524)  22616  (3285) | 170194  (25837)  18982  (2771) | 193857  (25606)  21728  (2958) | 176391  (26299)  19599  (2834) | 545085  (74721)  110883  (16103) |
| R_merge_ | 0.061 (0.947) | 0.047 (0.946) | 0.066 (1.137) | 0.051 (0.845) | 0.053 (0.862) | 0.045 (0.893) | 0.053 (0.804) | 0.047 (0.960) | 0.191 (1.405) |
| I/σ(I) | 18.0 (2.2) | 20.2 (2.4) | 14.8 (1.9) | 17.4 (2.1) | 17.8 (2.1) | 22.3 (2.2) | 19.1 (2.3) | 20.6 (2.1) | 5.4 (1.3) |
| CC(1/2) | 0.999 (0.866) | 0.999 (0.900) | 0.999 (0.754) | 0.999 (0.706) | 0.999 (0.808) | 0.999 (0.892) | 0.999 (0.839) | 0.999 (0.796) | 0.986 (0.465) |
| Completeness [%] | 100 (100) | 99.8 (100) | 99.7 (100) | 100 (100) | 100 (100) | 99.0 (100) | 98.3 (93) | 100 (100) | 99.9 (99.9) |
| Multiplicity | 10.3 (10.4) | 8.9 (9.1) | 8.6 (9.2) | 7.6 (7.9) | 8.9 (9.0) | 9.0 (9.3) | 8.9 (8.7) | 9.0 (9.3) | 4.9 (4.6) |
| **Refinement** |  |  |  |  |  |  |  |  |  |
| Refinement program | PHENIX | PHENIX | PHENIX | PHENIX | PHENIX | PHENIX | PHENIX | PHENIX | PHENIX |
| Resolution [Å] | 50.12 – 2.25 | 64.80 – 2.24 | 87.18 – 2.32 | 87.47 – 2.25 | 87.03 – 2.16 | 87.17 – 2.28 | 65.02 – 2.17 | 87.32 – 2.27 | 68.45 – 1.54 |
| No. reflections | 20440 | 20234 | 18318 | 20451 | 22616 | 18982 | 21728 | 19599 | 110292 |
| R_work_/R_free_ [%] | 19.0/25.1 | 19.5/23.6 | 21.1/24.3 | 19.2/23.9 | 19.1/23.8 | 19.3/22.5 | 18.8/23.6 | 18.9/23.3 | 19.3/21.2 |
| RMS deviations |  |  |  |  |  |  |  |  |  |
| Bonds [Å] | 0.008 | 0.009 | 0.009 | 0.009 | 0.009 | 0.008 | 0.009 | 0.009 | 0.010 |
| Angles [˚] | 0.982 | 1.111 | 1.100 | 1.021 | 0.975 | 0.982 | 0.965 | 1.037 | 1.36 |
| Ramachandran |  |  |  |  |  |  |  |  |  |
| Favoured [%] | 97 | 96 | 94 | 96 | 97 | 96 | 97 | 95 | 98.7 |
| Outliers [%] | 0.3 | 0.3 | 0.7 | 0.3 | 0.3 | 0.3 | 0.3 | 0.7 | 0 |

* Parameters shown in brackets are for the highest resolution shell

**Table S3:** X-ray crystallography data collection and final refinement statistics (cont)

| Protein | **ArgC** | **ArgC** | **ArgC** | **ArgC** | **ArgC** | **ArgD** | **ArgD** | **ArgD** | **ArgF** |
| --- | --- | --- | --- | --- | --- | --- | --- | --- | --- |
| Ligand# | **NADP** | **NMR322** | **NMR401** | **NMR571** | **NMR863** | **PLP** | **NMR608** | **NMR868** | **Apo** |
| PDB ID | 7NNQ | 7NOT | 7NPJ | 7NNR | 7NPH | 7NN1 | 7NN4 | 7NNC | 7NNF |
| **Data collection*** |  |  |  |  |  |  |  |  |  |
| Space group | *P2_1_* | *P2_1_* | *P2_1_* | *C2* | *P2_1_* | *P2_1_* | *P2_1_* | *P2_1_* | *P2_1_* |
| Cell parameters:  a [Å]  b [Å]  c [Å]  α/β/γ [˚] | 69.05  133.83  83.05  90/109.4/90 | 83.40  132.00  124.55  90/97.06/90 | 84.40  132.85  122.71  90/90.06/90 | 140.71  78.23  87.64  90/127.51/90 | 84.28  132.79  122.83  90/90.17/90 | 65.06  184.26  71.54  90/106.1/90 | 65.19  184.35  71.30  90/106.21/90 | 65.46  184.94  71.58  90/106.1/90 | 91.34  142.38  98.28  90/117.2/90 |
| Resolution range [Å] | 55.52 – 1.73  (1.77 – 1.73) | 63.91 – 2.54  (2.61 – 2.54) | 71.24 – 2.81  (2.88 – 2.81) | 69.51 – 1.7  (1.73 – 1.7) | 69.59 – 2.57  (2.64 – 2.57) | 62.50 – 1.54  (1.62 – 1.54) | 68.47 – 1.47  (1.51 – 1.47) | 68.78 – 1.70  (1.73 – 1.70) | 81.12 – 1.52 (1.61 – 1.52) |
| No. of observations  total  unique | 804727 (58410)  145458  (10386) | 463192 (36392)  87548  (6486) | 353903 (26408)  65900  (4807) | 417201  (15597)  81979  (3945) | 466260 (34746)  86043  (6338) | 1065135 (75420)  218441 (20903) | 1192624 (51854)  265823 (17510) | 948793 (41174)  177420 (8457) | 1731990 (209268)  338738  (49398) |
| R_merge_ | 0.048 (1.275) | 0.206 (2.135) | 0.117 (1.641) | 0.031 (0.091) | 0.067 (1.473) | 0.068 (0.873) | 0.035 (0.693) | 0.027 (0.082) | 0.096 (0.978) |
| I/σ(I) | 13.9 (1.0) | 7.3 (0.8) | 6.9 (1.0) | 29.3 (10.2) | 11.0 (1.0) | 9.4 (1.1) | 16.5 (1.2) | 34.0 (12.1) | 7.4 (1.0) |
| CC(1/2) | 0.999 (0.462) | 0.986 (0.317) | 0.993 (0.321) | 0.999 (0.988) | 0.999 (0.522) | 0.998 (0.438) | 0.999 (0.500) | 0.999 (0.994) | 0.994 (0.449) |
| Completeness [%] | 98.4 (95.4) | 99.3 (99.9) | 99.9 (100) | 99 (89.6) | 100 (100) | 91.7 (60.2) | 97.4 (86.7) | 99.3 (95.6) | 99.9 (100.0) |
| Multiplicity | 5.5 (5.6) | 5.3 (5.6) | 5.4 (5.5) | 5.1 (4) | 5.4 (5.5) | 4.9 (3.6) | 4.5 (3.0) | 5.3 (4.9) | 5.1 (4.2) |
| **Refinement** |  |  |  |  |  |  |  |  |  |
| Refinement program | PHENIX | PHENIX | PHENIX | PHENIX | PHENIX | PHENIX | PHENIX | PHENIX | PHENIX |
| Resolution [Å] | 45.09 – 1.73 | 58.44 – 2.54 | 69.46 – 2.81 | 40.57 – 1.7 | 69.4 – 2.57 | 46.81 – 1.539 | 64.18 – 1.47 | 45.91 – 1.70 | 81.12 – 1.52 |
| No. reflections | 145435 | 87492 | 65781 | 81962 | 85901 | 218262 | 265761 | 177364 | 338688 |
| R_work_/R_free_ [%] | 19.1/21.1 | 21.5/27.5 | 25.7/32.7 | 15.8/17.5 | 26/31.1 | 18/20 | 18/19.6 | 14.7/16.3 | 16.9/18.3 |
| RMS deviations |  |  |  |  |  |  |  |  |  |
| Bonds [Å] | 0.008 | 0.011 | 0.009 | 0.006 | 0.010 | 0.009 | 0.008 | 0.009 | 0.007 |
| Angles [˚] | 1.27 | 1.5 | 1.27 | 0.84 | 1.46 | 1.44 | 1.41 | 1.2 | 1.093 |
| Ramachandran |  |  |  |  |  |  |  |  |  |
| Favoured [%] | 97.9 | 96 | 86.99 | 98.8 | 93.1 | 97.6 | 97.60 | 97.5 | 98 |
| Outliers [%] | 0.07 | 0.3 | 2.08 | 0 | 0.7 | 0.2 | 0.3 | 0.4 | 0.4 |

* Parameters shown in brackets are for the highest resolution shell

**Table S3:** X-ray crystallography data collection and final refinement statistics (cont)

| Protein | **ArgF** | **ArgF** | **ArgF** | **ArgF** | **ArgF** | **ArgF** | **ArgF** | **ArgF** | **ArgF** |
| --- | --- | --- | --- | --- | --- | --- | --- | --- | --- |
| Ligand# | **CP** | **NMR007** | **NMR078** | **NMR288** | **NMR464** | **NMR502** | **NMR801** | **NMR812** | **NMR817** |
| PDB ID | 7NNV | 7NNZ | 7NNW | 7NNY | 7NOS | 7NOR | 7NP0 | 7NOU | 7NOV |
| **Data collection*** |  |  |  |  |  |  |  |  |  |
| Space group | *P2_1_* | *P2_1_* | *P2_1_* | *P2_1_* | *P2_1_* | *P2_1_* | *P2_1_* | *P2_1_* | *P2_1_* |
| Cell parameters:  a [Å]  b [Å]  c [Å]  α/β/γ [˚] | 91.44  142.37  99.02  90/117.2/90 | 91.62  143.00  97.59  90/117.6/90 | 91.80  143.51  97.52  90/117.7/90 | 91.95  143.79  97.28  90/117.6/90 | 92.00  143.61  97.82  90/117.5/90 | 91.45  142.73  97.70  90/117.5/90 | 92.04  143.45  97.53  90/117.5/90 | 92.30  144.66  97.76  90/117.8/90 | 92.26  144.42  97.35  90/117.8/90 |
| Resolution range [Å] | 88.10 – 1.67  (1.76 – 1.67) | 81.20 – 1.68  (1.77 – 1.68) | 86.36 – 1.78  (1.87 – 1.78) | 86.21 – 1.57  (1.66 – 1.57) | 81.61 – 1.77  (1.86 – 1.77) | 81.12 – 1.59  (1.68 – 1.59) | 70.46 – 1.76  (1.85 – 1.76) | 86.47 – 1.98  (2.08 – 1.98) | 86.15 – 1.90  (2.00 – 1.90) |
| No. of observations  total  unique | 1330397 (151816)  259225 (36285) | 1305874 (162377)  252690 (36825) | 1131278 (166972)  214281  (31302) | 1448621 (105414)  294032 (31609) | 1170700 (168321)  219906 (32044) | 1416962 (114917)  271697 (26818) | 1149709 (167284)  222029 (32413) | 1604954 (230134)  157930 (23055) | 840668 (80109)  170994 (20551) |
| R_merge_ | 0.051 (1.051) | 0.089 (0.898) | 0.078 (1.140) | 0.043 (0.937) | 0.045 (1.140) | 0.052 (1.109) | 0.047 (0.508) | 0.083 (0.895) | 0.057 (0.312) |
| I/σ(I) | 13.6 (0.9) | 9.3 (1.7) | 10.9 (1.1) | 15.4 (0.9) | 15.5 (1.1) | 13.0 (0.9) | 16.0 (2.7) | 16.1 (2.7) | 14.1 (2.6) |
| CC(1/2) | 0.999 (0.460) | 0.997 (0.456) | 0.999 (0.530) | 0.999 (0.424) | 0.999 (0.551) | 0.999 (0.427) | 0.999 (0.801) | 0.998 (0.830) | 0.998 (0.884) |
| Completeness [%] | 99.2 (95.2) | 99.9 (99.8) | 99.9 (100) | 94.6 (69.8) | 100 (100) | 92.2 (62.3) | 99.9 (100) | 99.9 (100) | 96.7 (79.6) |
| Multiplicity | 5.1 (4.2) | 5.2 (4.4) | 5.3 (5.3) | 4.9 (3.3) | 5.3 (5.3) | 5.2 (4.3) | 5.2 (5.2) | 10.2 (10.0) | 4.9 (3.9) |
| **Refinement** |  |  |  |  |  |  |  |  |  |
| Refinement program | PHENIX | PHENIX | PHENIX | PHENIX | PHENIX | PHENIX | PHENIX | PHENIX | PHENIX |
| Resolution [Å] | 88.10 – 1.67 | 81.20 – 1.68 | 86.36 – 1.78 | 86.21 – 1.57 | 81.61 – 1.77 | 81.12 – 1.59 | 70.46 – 1.76 | 86.47 – 1.98 | 86.15 – 1.90 |
| No. reflections | 259158 | 252638 | 423145 | 293962 | 219865 | 271656 | 221982 | 157873 | 170957 |
| R_work_/R_free_ [%] | 16.3/18.0 | 16.7/18.3 | 18.1/20.6 | 16.6/18.4 | 17.0/19.2 | 16.7/18.6 | 16.2/18.2 | 17.4/20.1 | 16.1/19.0 |
| RMS deviations |  |  |  |  |  |  |  |  |  |
| Bonds [Å] | 0.009 | 0.010 | 0.009 | 0.007 | 0.012 | 0.008 | 0.007 | 0.011 | 0.009 |
| Angles [˚] | 1.463 | 1.622 | 1.419 | 1.213 | 1.596 | 1.177 | 0.959 | 1.572 | 1.483 |
| Ramachandran |  |  |  |  |  |  |  |  |  |
| Favoured [%] | 97 | 97 | 97 | 98 | 97 | 98 | 97 | 97 | 97 |
| Outliers [%] | 0.4 | 0.4 | 0.3 | 0.3 | 0.4 | 0.4 | 0.3 | 0.7 | 0.4 |

* Parameters shown in brackets are for the highest resolution shell

**References**

1. Sievers F, Wilm A, Dineen D, Gibson TJ, Karplus K, Li W, Lopez R, McWilliam H, Remmert M, Soding J, Thompson JD, Higgins DG. 2011. Fast, scalable generation of high-quality protein multiple sequence alignments using Clustal Omega. Mol Syst Biol 7:539.
